# Efficacy of ultrasound-guided intra-articular injection in the treatment of knee osteoarthritis in early and middle stages: a network meta-analysis

**DOI:** 10.3389/fmed.2025.1700950

**Published:** 2025-11-06

**Authors:** Jiahao Zhang, Yuqi Guo, Bowen Lu, Gang Li, Jiacheng Li

**Affiliations:** 1 Shandong University of Traditional Chinese Medicine, Shandong, China; 2 Affiliated Hospital of Shandong University of Traditional Chinese Medicine, Shandong, China

**Keywords:** knee osteoarthritis, intra-articular injection, ultrasound, platelet-rich plasma, hyaluronic acid, Bayesian network meta-analysis

## Abstract

**Background:**

Knee osteoarthritis (KOA) is a prevalent degenerative joint disorder causing significant pain and functional impairment. Intra-articular injections (IAI) under ultrasound (US) guidance have emerged as a key treatment for early-to-mid stage KOA due to enhanced precision and localized therapeutic effects. However, the relative efficacy of various injectable agents remains unclear.

**Methods:**

A Bayesian network meta-analysis (BNMA) was conducted following PRISMA guidelines using the following databases: ClinicalTrials.gov, EMBASE, PubMed, Web of Science, Cochrane Library, and the WHO International Clinical Trials Registry Platform (WHO ICTRP), we searched incorporating 14 randomized controlled trials (RCTs) involving 934 patients. Interventions included US-guided injections of platelet-rich plasma (PRP), hyaluronic acid (HA), corticosteroids (CS), ozone (O3), dexamethasone (DX), autologous adipose tissue (AAT), and placebo (PL). Primary outcomes were Visual Analog Scale (VAS) and WOMAC subscale scores. Model consistency, transitivity, and robustness were rigorously assessed.

**Results:**

The analysis demonstrated that all active interventions provided significant symptom relief. PRP consistently ranked highest across multiple outcomes, with surface under the cumulative ranking curve (SUCRA) values of 85.86% for total WOMAC score, 78.55% for pain, 93.24% for stiffness, and 90.9% for function. HA showed significant superiority over ozone in pain reduction (SMD: −1.48, 95% CI: −2.71 to −0.24). Model consistency was confirmed (*p* > 0.05 for all node-splitting tests), and sensitivity analyses supported result stability. No significant publication bias was detected.

**Conclusion:**

IAI under US-guided has shown good therapeutic effect in the treatment of KOA in the early and middle stages, and PRP has been proved to have the highest therapeutic possibility, followed by HA and CS. These findings support the use of US-guided biologic interventions as part of a comprehensive KOA management strategy, though standardization of protocols and long-term outcomes require further investigation.

## 1 Introduction

Knee osteoarthritis (KOA) is a chronic joint disorder characterized by primary pathological features including degenerative changes in articular cartilage, subchondral bone sclerosis, osteophyte formation, and synovitis (1). As the most prevalent musculoskeletal disorder causing pain and functional impairment worldwide, KOA not only severely impacts patients' quality of life but also imposes substantial economic burdens on healthcare systems (2, 3). With the acceleration of global population aging and rising obesity rates (4, 5), KOA affects over 500 million people worldwide (6), with ~10%−15% of individuals aged 60 and above suffering from symptomatic KOA. The pathophysiology of KOA involves interactions among multiple factors including abnormal mechanical loading, activation of inflammatory responses, and imbalance in cartilage metabolism. These ultimately lead to joint structural damage, subsequently triggering clinical manifestations such as joint pain, stiffness, and functional impairment (7). According to the Kellgren–Lawrence radiographic grading system, KOA can be classified into four stages. Stages I to II represent early to mid-stage disease, characterized by suspected or definite joint space narrowing and mild osteophyte formation; stages III–IV represent advanced disease, characterized by significant joint space narrowing, subchondral bone sclerosis, and formation of large osteophytes (8). Clinical treatment strategies should follow a stepped approach tailored to individual patients based on disease stage. Due to discrepancies between radiographic findings and clinical manifestations, a subset of Stage III patients also meet the treatment indications for early-to-mid stage disease and are therefore categorized as having “mild-to-moderate KOA”.

For advanced KOA patients, total knee arthroplasty (TKA) is considered an effective intervention for pain relief and functional restoration (9). However, TKA is traumatic, costly and carries risks of complications including prosthetic loosening and infection. Therefore, clinical guidelines widely recommend that non-surgical treatments should be pursued for several years before considering TKA. These conservative treatments include oral non-steroidal anti-inflammatory drugs (NSAIDs) (10), analgesic medications such as acetaminophen, intra-articular injection (IAI) therapy, as well as comprehensive interventions like physical therapy, exercise programs, and weight management (11). Among these, intra-articular injections have become an important treatment option for early to mid-stage KOA due to their strong localized effects and minimal systemic side effects. Currently, commonly used intra-articular injectables in clinical practice primarily include hyaluronic acid (HA) (12), glucocorticoids (CS) (13), platelet-rich plasma (PRP) (14) and various stem cell preparations. However, the accuracy of intra-articular injection techniques directly impacts therapeutic efficacy. Anatomical studies reveal that knee joint cavity injections achieve an accuracy rate of only ~70% when performed as “blind punctures” without imaging guidance (15). In severe KOA patients with significant joint deformity, the injection accuracy rate further decreases due to osteophyte proliferation, joint space narrowing, and alterations in surface anatomical landmarks. Inaccurate injections may lead to abnormal drug distribution, reduced therapeutic efficacy, and even increased risk of local tissue damage (16, 17). Image-guided techniques have been gradually adopted in clinical practice to improve injection precision. Among these, ultrasound (US) guidance has gained widespread attention due to its advantages of being radiation-free, providing real-time dynamic imaging, and offering portability with user-friendly operation (18).

In recent years, multiple studies have investigated the impact of US guidance on the efficacy of intra-articular injections (19, 20). Systematic reviews demonstrate that compared with traditional landmark-based techniques, US guidance significantly improves injection accuracy rates. Theoretically, precise drug delivery should enhance local bioavailability and improve clinical efficacy. However, existing evidence exhibits significant heterogeneity: some studies found US-guided injections substantially enhanced the efficacy of HA and CS (21), while others observed no significant differences (22, 23). These inconsistencies may stem from variations in study design, patient selection, injection techniques, and outcome assessments. Furthermore, systematic evaluations are currently lacking regarding: (1) relative efficacy differences among injectable agents under US guidance, and (2) whether US-mediated therapeutic enhancement varies across different injectables.

Traditional pairwise meta-analysis methods struggle to simultaneously compare multiple interventional approaches. In contrast, Bayesian Network Meta-Analysis (BNMA), as an advanced statistical technique, integrates both direct and indirect comparative evidence, enabling the evaluation of the relative efficacy and safety profiles of multiple interventions within a unified framework. Against this background, this study employs BNMA to systematically evaluate the efficacy and safety of different US-guided intra-articular injection agents for treating early-to-mid stage KOA. This study aims to clarify the relative efficacy and safety profiles of different interventions, thereby providing a high-quality evidence base for optimizing clinical treatment strategies, enhancing knee preservation therapy outcomes, and improving the quality of life and functional prognosis of KOA patients.

## 2 Materials and methods

This study was conducted in strict adherence to the latest PRISMA guidelines and relevant protocol specifications, with prior registration in the PROSPERO database (Registration Number: CRD420251037290).

### 2.1 Literature search

We systematically searched six English databases and clinical trial registries including ClinicalTrials.gov, EMBASE, PubMed, Web of Science, Cochrane Library, and the WHO International Clinical Trials Registry Platform (WHO ICTRP) to identify clinical studies on US-guided intra-articular injection (US+IAI) therapy for early to mid-stage KOA. The search encompassed records from database inception through January 22, 2025, utilizing a combination of subject headings and free-text terms. Search terms included “Osteoarthritis, Knee”, “knee osteoarthritis”, “Ultrasonography”, “US-guided”, “Injections”, and “randomized controlled trial”. Logical expressions were constructed using MeSH terms combined with keywords.

### 2.2 Inclusion criteria

- Study type: randomized controlled trials (RCTs)
- Interventional approaches: for two-arm studies, both groups must receive IAI interventions, with at least one group undergoing US+IAI. In multi-arm studies, at least two groups must receive IAI interventions, with at least one group undergoing US+IAI. Medication types used for injections were not restricted.
- Study subjects: (1) human participants; (2) participants must present with radiological or clinical evidence of KOA. Unified inclusion criteria shall follow authoritative references such as the American College of Rheumatology diagnostic criteria for KOA, selecting patients with Kellgren-Lawrence (K-L) grade I-III or those explicitly defined as “mild-to-moderate KOA” in literature with radiologically confirmed staging.
- Outcome measures: included studies must report at least one of the following outcome measures: Visual Analog Scale (VAS), used to assess patients' subjective pain intensity; WOMAC Pain Subscale (WOMAC-P): Quantifies the intensity of joint pain experienced during daily activities; WOMAC Function Subscale (WOMAC-F): Assesses the impact of functional impairment on activities of daily living; WOMAC Stiffness Subscale (WOMAC-S): Measures the subjective severity and duration of joint stiffness; WOMAC total score (WOMAC-T): comprehensively reflects overall joint status by integrating pain, stiffness, and functional impairment.

To control for potential statistical bias introduced by inconsistent scoring directions, all outcome measures underwent positive standardization during data extraction and organization. This process uniformly converted all measures to the direction where “higher values indicate better efficacy,” ensuring consistency in effect size estimation and enhancing comparability of outcome measures across different studies.

### 2.3 Exclusion criteria

- Non-RCTs, conference abstracts, animal studies, and studies with inaccessible full texts.
- Articles with duplicated data, significant design flaws, or missing critical outcome data.
- Studies where participants did not meet clinical diagnostic or staging criteria for early to mid-stage KOA.
- Non-IAI approaches or those lacking US guidance

### 2.4 Literature screening and quality assessment

Two reviewers (Zhang, Guo) independently conducted the evaluations, subsequently cross-verifying the assessment outcomes. Discrepancies in evaluations were resolved through adjudication by a third reviewer to ensure objectivity and consistency.

The methodological quality of included studies was systematically evaluated using the Cochrane Risk of Bias tool, assessing five domains: bias in randomization process, deviations from intended interventions, missing outcome data, measurement of outcomes, and selective reporting. The RoB 2.0 tool systematically evaluates the risk of bias in each study through seven specific questions, classifying studies into three tiers-“Low risk,” “High risk,” or “Some concerns”-based on Cochrane-recommended standards. Assessments strictly adhered to this framework to ensure highly credible evaluation of methodological quality in the literature while meeting fundamental requirements of evidence-based medicine.

### 2.5 Statistical analysis

Network meta-analysis was performed using a Bayesian model. Statistical analyses were conducted in R software (version 4.4.3) utilizing packages including gemtc, rjags, netmeta, dmetar, and BUGSnet. All outcome measures were continuous variables, with effect sizes expressed as Standardized Mean Difference (SMD) and 95% Confidence Interval (CI). This approach ensured comparability across studies despite variations in measurement tools and units.

The evidence network structure was constructed via the mtc.network function, with node size representing the total sample size of interventional approaches and edge thickness reflecting the number of direct comparison studies, thereby visualizing network relationships and evidence strength among interventions. Model construction employed the mtc.model function to establish consistency structures and random-effects parameters. Four distinct models were systematically developed: consistency+ fixed effects, inconsistency + fixed effects, consistency + random effects, and inconsistency + random effects. These were evaluated for model fitting performance and heterogeneity control to identify the optimal model. Posterior sampling was executed using JAGS with four Markov chains (50,000 burn-in iterations+100,000 sampling iterations) and a thinning interval of 1. Model performance evaluation metrics included the Deviance Information Criterion (DIC) and Potential Scale Reduction Factor (PSRF). The DIC comprehensively measures model fit and complexity, while a PSRF ≤ 1.05 indicates that the model has met convergence criteria.

The analysis workflow comprised consistency testing, transitivity assessment, BNMA, Surface Under the Cumulative Ranking Curve (SUCRA) ranking, sensitivity analysis, and publication bias evaluation. Local inconsistency testing employed the Node-Splitting Method for intervention pairs with both direct and indirect comparison pathways. This method analyzed differences between estimated effects and presented direct effects, indirect effects, and network effects through forest plots. Transitivity hypothesis testing was conducted based on baseline characteristics of intervention cohorts (mean age, gender ratio, injection frequency), with distributional balance of covariates presented through composite figures. Following consistency testing and transitivity assessment, BNMA was implemented, and an enhanced league plot was generated to visualize pairwise comparisons between interventions. The SUCRA ranking demonstrated relative superiority probabilities of interventional approaches, while cumulative probability curves and rankograms visualized ranking distributions across different outcome measures. For the primary outcome measure, sensitivity analysis using the stepwise elimination method evaluated individual study influence on effect estimates, with results displayed in heatmaps. Publication bias was assessed via adjusted funnel plots and Egger's regression test. The statistical analysis plan was reviewed and approved by biostatistics experts at Shandong University of Traditional Chinese Medicine, ensuring scientific rigor and methodological validity.

## 3 Results

### 3.1 Literature retrieval

**Figure 1:**
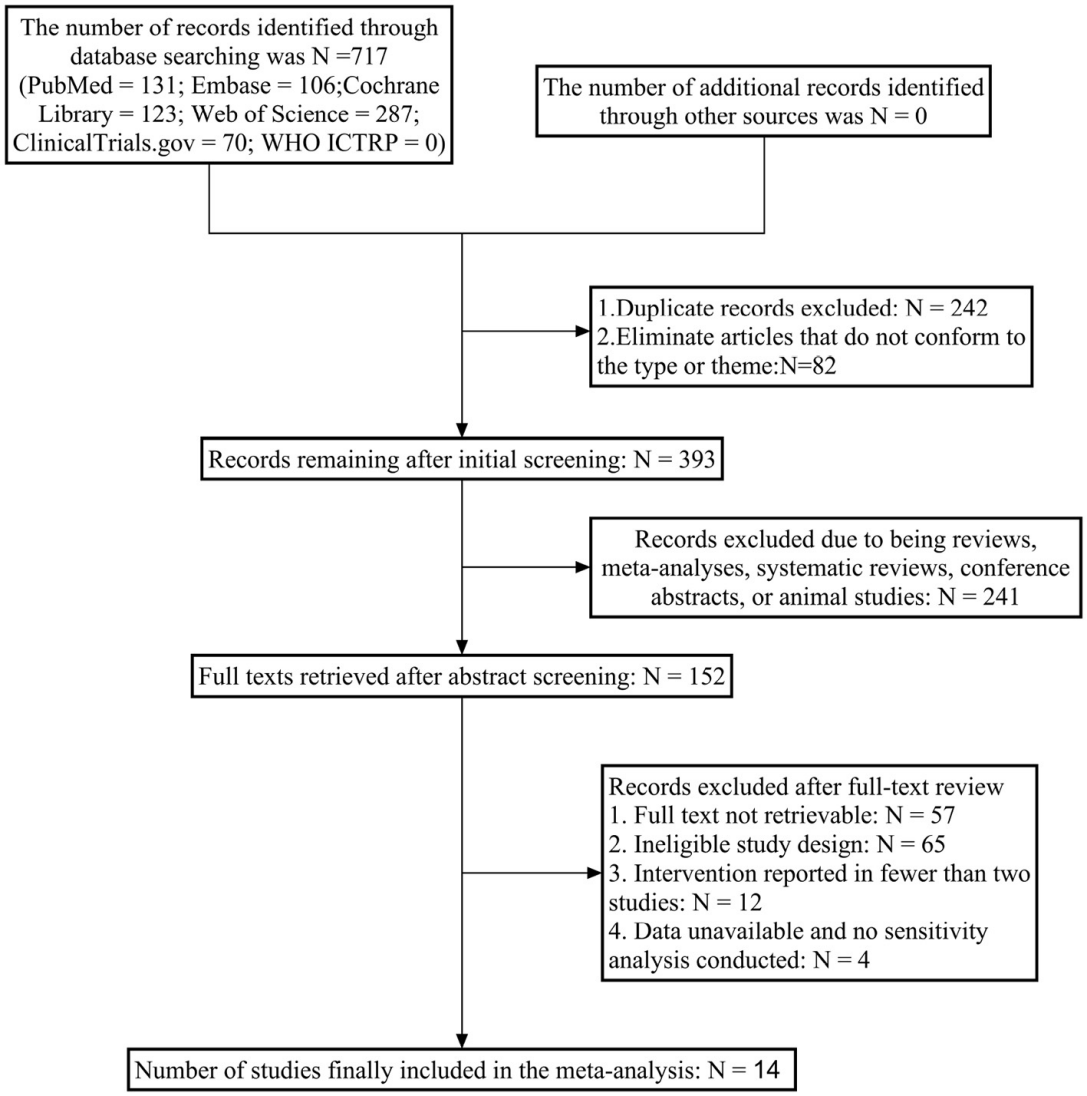
Literature search flowchart.

Initial searches across six English databases yielded 717 relevant articles. After removing 242 duplicates and excluding 82 articles with mismatched types or topics, 393 articles underwent preliminary screening. Following abstract screening, 241 articles (including animal studies, meta-analyses, and reviews) were excluded, resulting in 152 articles for further assessment. After full-text review, 138 articles were excluded due to unavailability of full texts, mismatched study designs, unusable data, or inability to construct outcome measures. Ultimately, 14 RCT (24–36) articles were included for network meta-analysis. The literature screening process is illustrated in Figure 1.

### 3.2 Basic characteristics of included literature

The 14 included RCTs involved 934 patients (intervention group: 468, control group: 466). All interventional approaches employed IAI therapies using US-guided localization combined with various agents, encompassing seven strategies: US+PL (3 studies), US+HA (4 studies), US+CS (5 studies), US+PRP (9 studies), US+O _3_ (3 studies), US+DX (2 studies), and US+AAT (2 studies). The included literature was published between 2017 and 2025. Basic characteristics of the included studies are presented in Supplementary Table 1.

### 3.3 Quality assessment of included studies

The Cochrane-recommended RoB 2.0 tool was used to assess the risk of bias in the 14 included RCTs. Regarding the randomization process, 7 studies employed computer- or software-assisted randomization methods, 5 used block randomization or other simple non-computer-generated randomization techniques, and 1 combined random sequence generation with sealed envelope allocation-all were rated as low risk of bias; One study failed to clearly report the specific randomization method and was assessed as having some concerns regarding risk of bias. Regarding blinding implementation, three studies adopted a single-blind design, seven utilized a double-blind design, and one employed a triple-blind design; all these studies were rated as having a low risk of bias. One study did not mention blinding implementation, while two other studies did not implement blinding but this did not affect the measurement and inference of primary outcomes; both were rated as having some risk. Regarding allocation concealment, seven studies clearly implemented allocation concealment measures and were rated as having a low risk of bias; the remaining seven studies did not report relevant measures and were rated as having some risk of bias. Regarding outcome data and reporting completeness, all studies provided complete outcome data without exclusions or dropouts, and reported statistical significance for all measures, thus being rated as low risk of bias.

**Figure 2:**
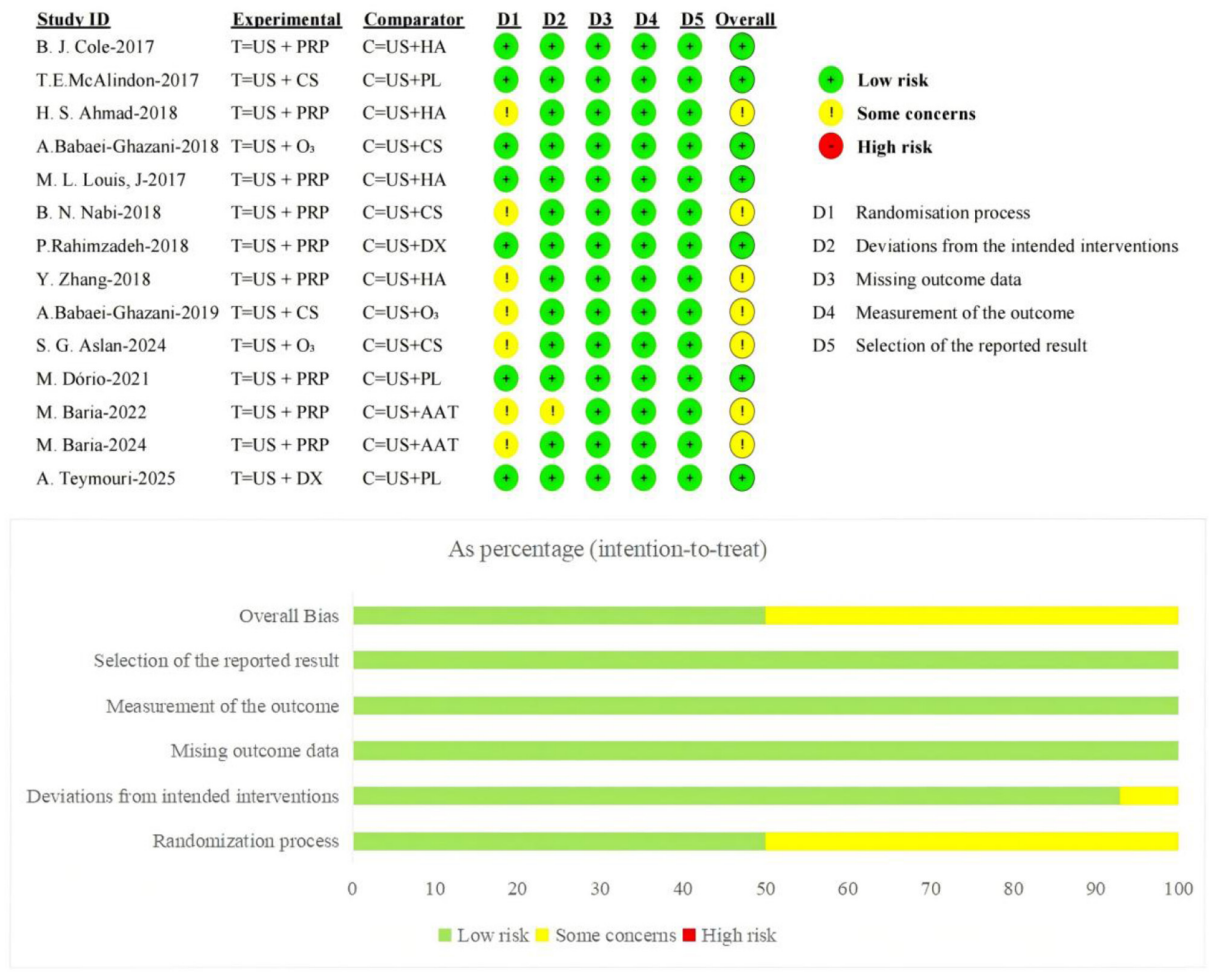
Risk of bias distribution.

Furthermore, all studies employed appropriate measurement methods to assess outcome measures. No selective outcome reporting bias was identified, with relevant aspects assessed as low risk of bias. Overall, the included studies demonstrated high methodological quality. The primary risk of bias stemmed from insufficient reporting of randomization procedures and blinding implementation details. The distribution and assessment results for each bias domain are presented in Figure 2.

### 3.4 Evidence network and model selection

**Figure 3:**
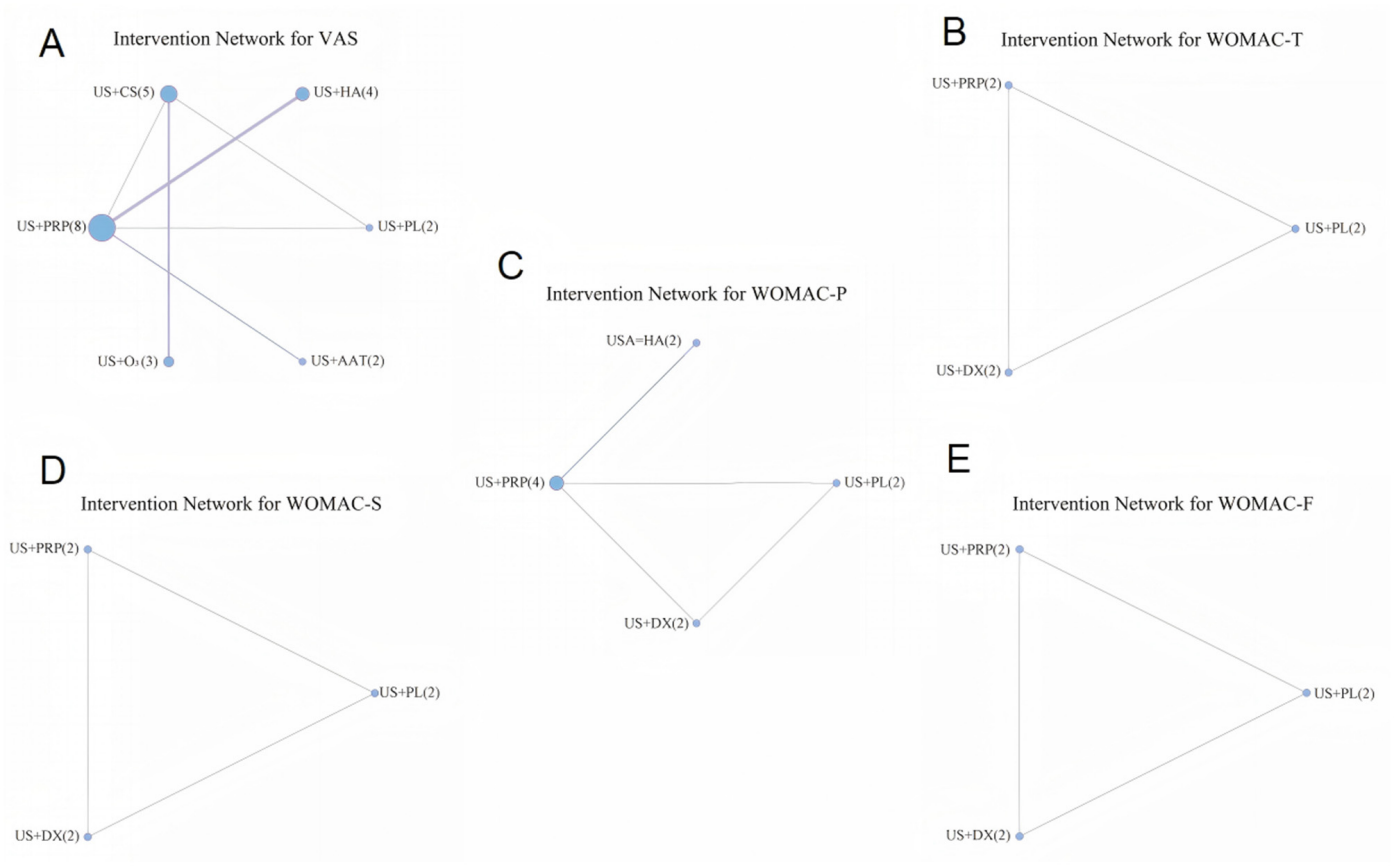
Evidence network diagrams for different outcome indicators (**A**: VAS, **B**: WOMAC-T, **C**: WOMAC-P, **D**: WOMAC-S, **E**: WOMAC-F).

Figure 3 displays the evidence networks constructed for seven IAI across five Efficacy Outcome Measures. Each node represents an interventional approach, with node size corresponding to the sample size of included studies. Line thickness indicates the number of direct comparison studies between two interventions, while the numeral following each intervention denotes the number of included studies for that specific outcome measure.

Comprehensive evaluation of model goodness-of-fit and heterogeneity levels determined the suitability under different model specifications. Supplementary Table 2 summarizes the fitting results for five outcome measures under both fixed-effect and random-effect models, comparing consistency and inconsistency models. Results demonstrated that across all outcome measures, the consistency model with random effects exhibited optimal performance in both model fitting and heterogeneity control, thus being established as the definitive analytical model for subsequent BNMA.

### 3.5 Consistency assessment

**Figure 4:**
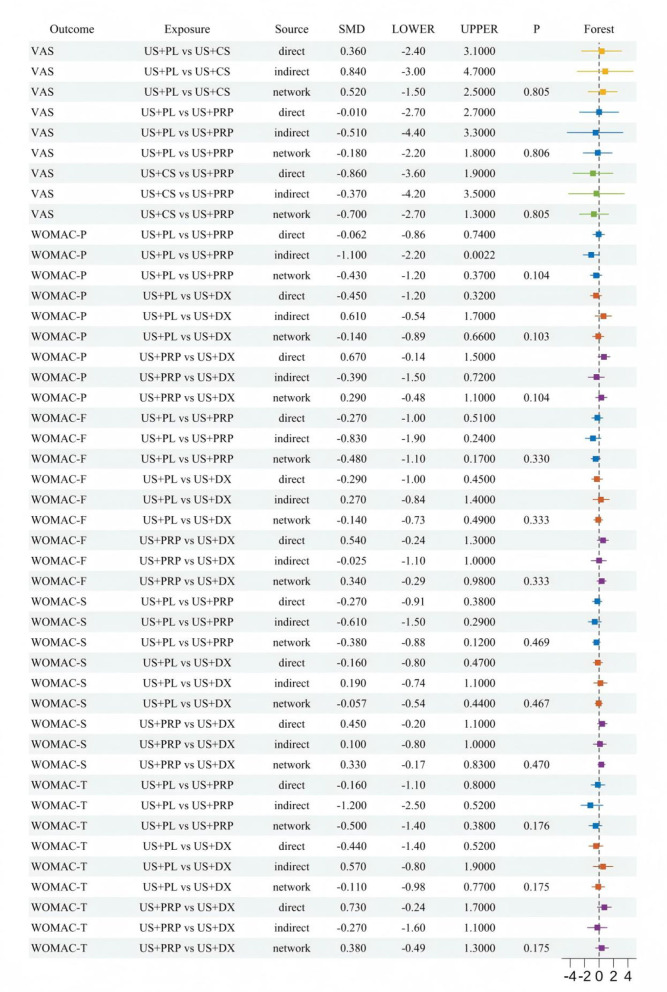
Forest map of inconsistent test results.

Node-splitting methodology was employed to conduct inconsistency tests on all comparable intervention pairs, evaluating agreement between direct and indirect evidence within the network. Multiple intervention comparisons encompassing five efficacy outcome measures were incorporated. As illustrated in Figure 4, all comparative *p*-values exceeded 0.05, with no statistically significant inconsistency observed, indicating robust applicability of the consistency model in this network analysis.

### 3.6 Transitivity assessment

**Figure 5:**
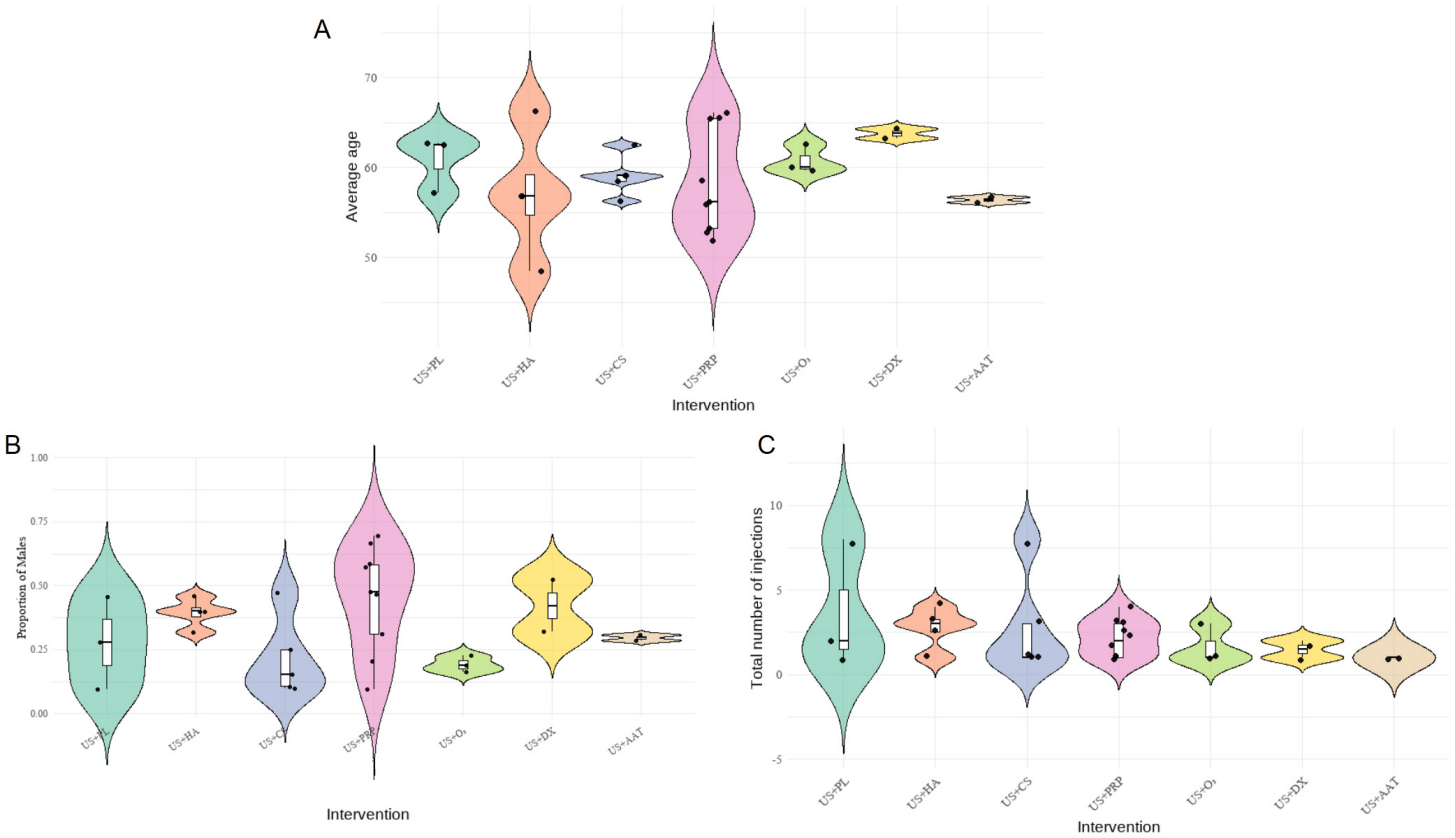
Composite distribution map of baseline characteristics of each intervention group (**A**: average age, **B**: gender ratio, **C**: injection frequency).

To evaluate potential systematic bias in indirect comparisons, a transitivity assessment was performed on three key effect modifiers affecting outcomes: age, gender, and number of injections, as shown in Figure 5. A composite figure integrating violin plots, box plots, and scatter plots was employed to visualize their distributional characteristics. Results demonstrated that male proportions across interventions ranged from 25% to 75%, mean ages generally distributed between 50 to 65 years, and total injection frequencies were predominantly concentrated within 1 to 5 administrations. The overall distributions exhibited no abnormal trends and did not constitute systematic bias. The transitivity assumption was validated.

### 3.7 Network meta-analysis

**Figure 6:**
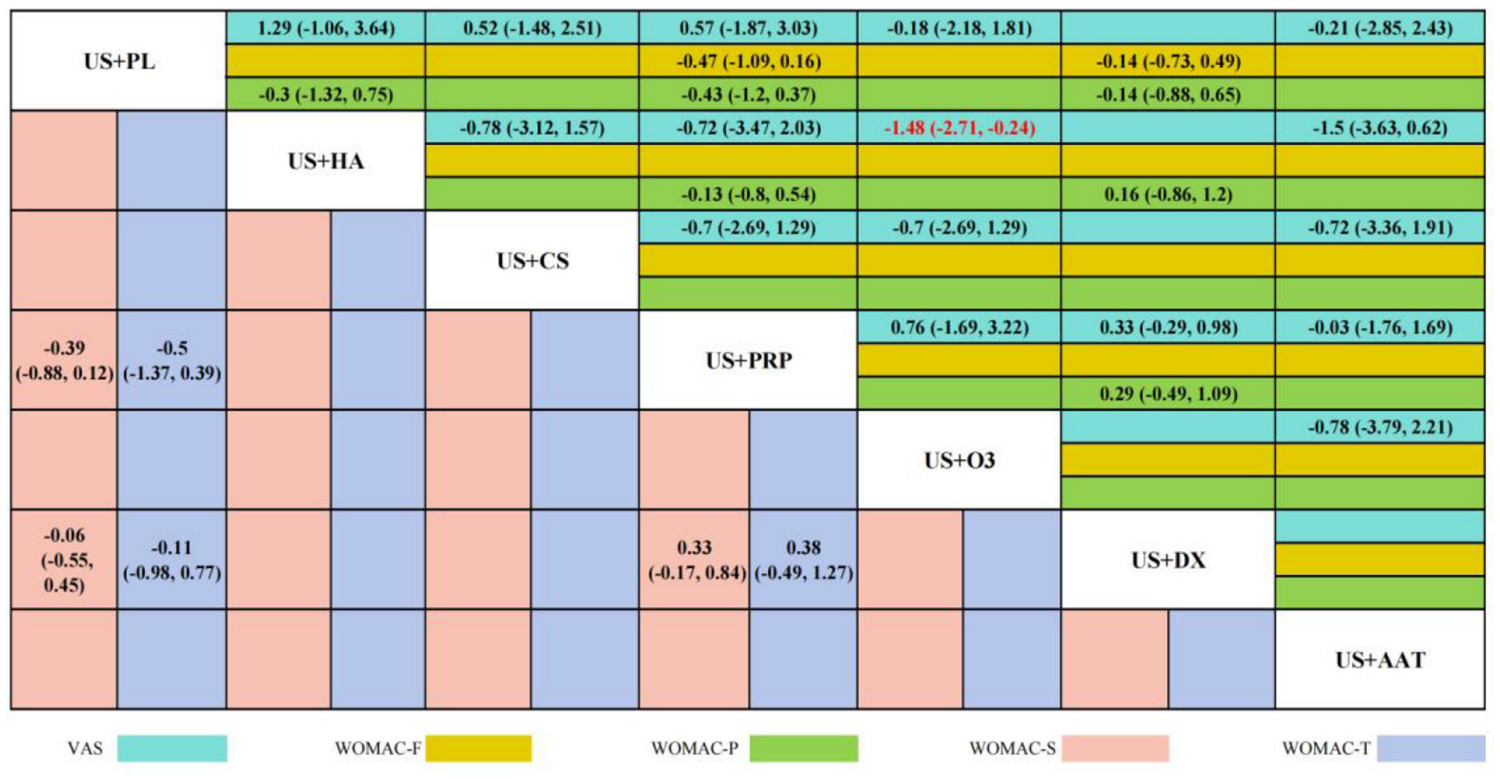
Enhanced alliance table of intervention measures under multiple outcome indicators.

Twelve RCTs reported VAS scores involving seven IAI. BNMA demonstrated that only US+O_3_ vs. US+HA [SMD = −1.48 (−2.71, −0.24), *p* < 0.05] reached statistical significance, indicating superior efficacy of US+HA over US+O_3_ in pain alleviation. Although no statistical differences were observed among other intervention cohorts, Five RCTs reported WOMAC-P scores, three RCTs reported WOMAC-F scores, three RCTs reported WOMAC-S scores, and three RCTs reported WOMAC-T scores. These involved four interventional approaches: US+DX (dexamethasone), US+PL (placebo), US+PRP (platelet-rich plasma), and US+HA (hyaluronic acid). BNMA results indicated statistical significance exclusively in the US+HA vs. US+O_3_ comparison [−1.48 (−2.71, −0.24)], suggesting HA's superior efficacy over O_3_ in alleviating pain. No statistical significance was demonstrated in the between-group analyses of all other outcome measures. However, consistent SMD directions were observed across most comparisons. The overall trend indicated a potential advantage of US+PRP across multiple WOMAC dimensions, particularly in WOMAC-Pain [−0.47 (−1.09, 0.16)] and WOMAC-Stiffness [−0.33 (−0.84, 0.17)], where notable effect sizes emerged. Nevertheless, the confidence intervals spanning zero imply limited reliability of these findings. The wide confidence intervals in comparisons between other interventional approaches further limit the reliability of the results. Detailed pairwise comparison outcomes are presented in Figure 6.

### 3.8 SUCRA ranking

**Figure 7:**
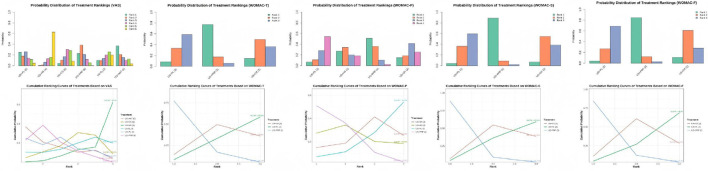
Rank distribution of interventions and cumulative probability analysis plot based on the SUCRA for each outcome measure.

We generated the cumulative SUCRA ranking curves and probability distribution diagrams of rank positions for interventions based on five outcome measures, as shown below. Performance variations existed among interventions across different outcome measures. US+PRP demonstrated higher cumulative ranking probabilities in most indicators, suggesting it may yield optimal effectiveness in pain relief and functional improvement. Regarding VAS, US+AAT exhibited the best intervention effect, followed by US+PRP and US+PL, indicating all three approaches effectively alleviated pain. Regarding WOMAC outcomes, US+PRP demonstrated the optimal interventional efficacy across all WOMAC parameters, indicating its superiority over other approaches in alleviating patient pain, improving knee function, and reducing stiffness severity (Figure 7).

### 3.9 Sensitivity analysis

**Figure 8:**
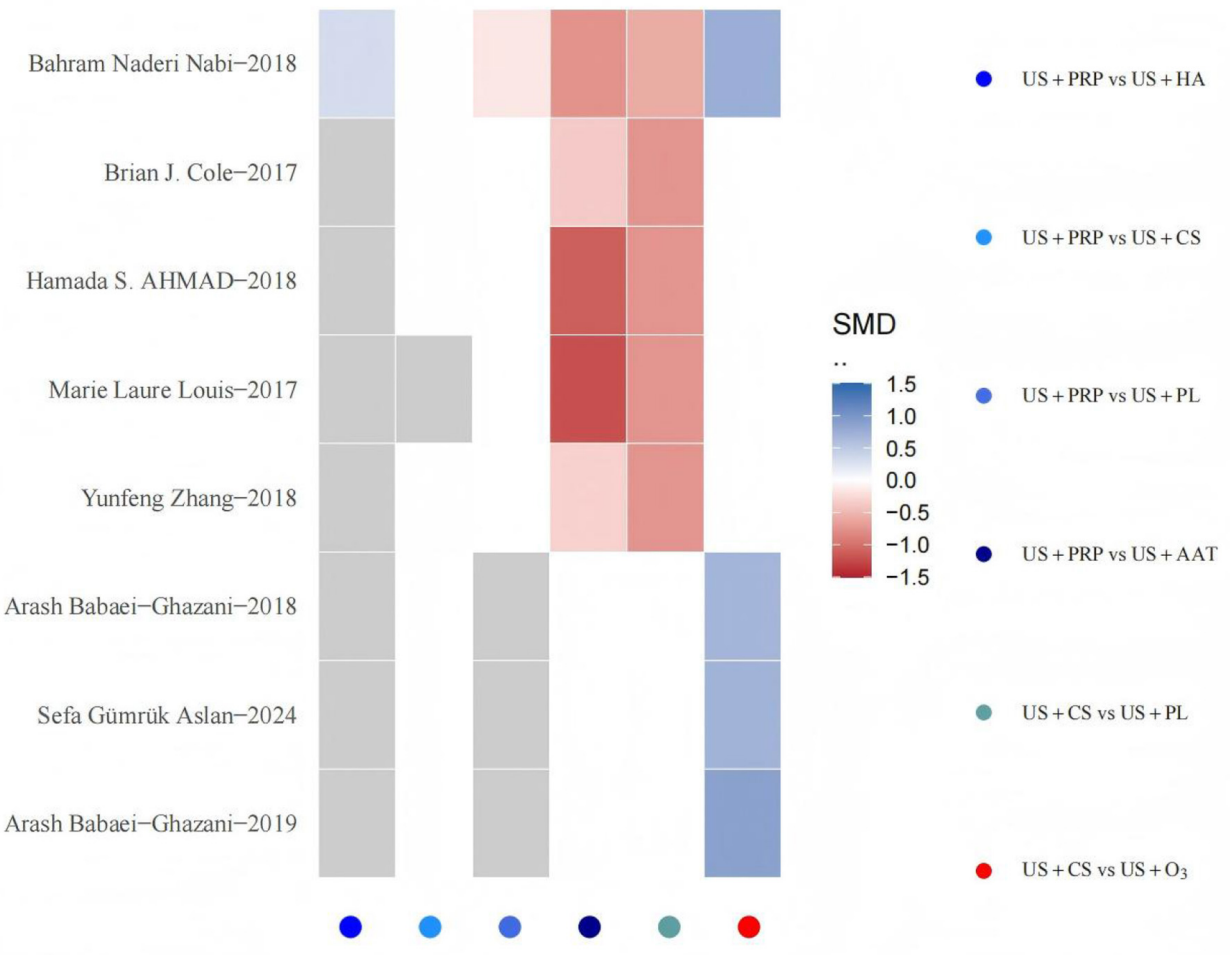
Sensitivity analysis heatmap. The horizontal axis represents all pairwise comparison combinations of interventional approaches (identified by color legend), while the vertical axis lists individual studies excluded sequentially. Each grid signifies the direction and magnitude of SMD changes for specific intervention comparisons after removing the corresponding study. Gray indicates that the intervention comparison persists after study exclusion but exhibits zero SMD change, whereas white denotes that the intervention comparison disappears from the network following study removal. The color gradient from blue to red reflects the transition of SMD changes from decrease to increase, with deeper hues indicating greater magnitude of change. Values approaching absolute zero appear closer to white.

To evaluate the robustness of findings, sensitivity analyses were performed for interventional approaches with at least three included studies. The stepwise elimination method was employed to quantitatively assess individual studies' impact on overall effects, observing directional changes and magnitude variations in SMDs and 95% CIs between intervention pairs. Results indicated that exclusion of any single study did not cause substantial changes in effect estimates or statistical significance, suggesting strong structural stability of the model. Specifically, the effect estimates for US+PRP interventional approaches remained relatively stable after sequential exclusion of multiple studies, further demonstrating the model's robustness against interference under this outcome measure. Following exclusion of studies such as “Ahmad et al. (24)” and “Louis et al. (29)”, although the SMD variation for US+HA interventional pairs exceeded ±1, the significance level showed no substantial alteration and did not constitute conclusive interference. The sensitivity analysis results demonstrate that the BNMA model exhibits high stability (Figure 8).

### 3.10 Publication bias

**Figure 9:**
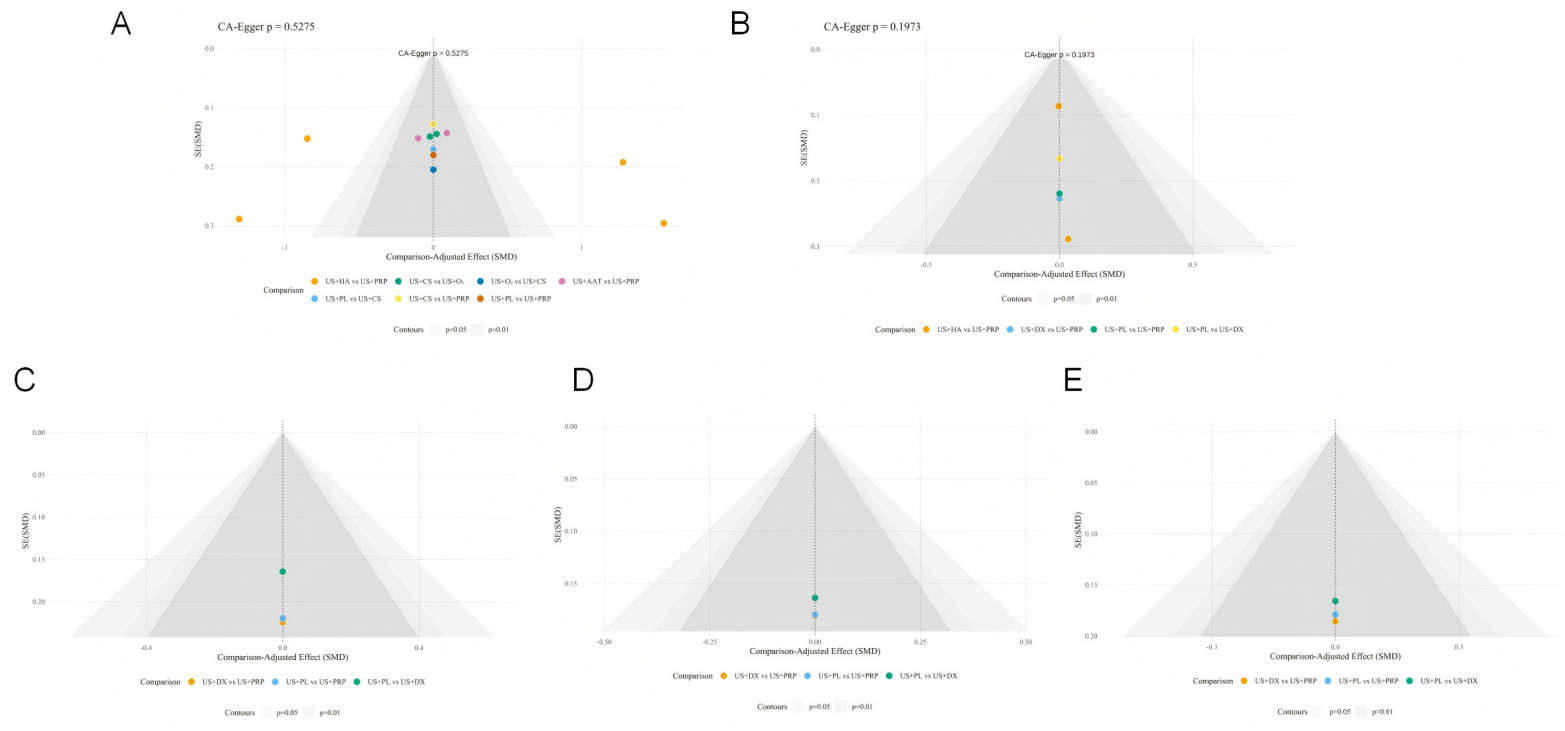
Corrected funnel plots across different outcome measures (**A**: VAS, **B**: WOMAC-T, **C**: WOMAC-P, **D**: WOMAC-S, **E**: WOMAC-F).

This study systematically evaluated publication bias for all outcome measures using Egger's regression test and comparative adjusted funnel plots in Figure 9. The adjusted funnel plot from Egger's test for the primary efficacy outcome showed symmetrically distributed study points (*p* > 0.05), indicating no significant publication bias. Due to the limited number of included studies, corresponding p-values could not be calculated for some assessments; however, no obvious publication bias was observed according to the adjusted funnel plots.

## 4 Discussion

This study incorporated 14 RCTs and systematically evaluated the comprehensive efficacy of multiple US-guided IAI therapies for early-to-mid stage KOA through outcome measures including VAS and WOMAC scores using NMA methodology. Through inconsistency tests and transitivity assessment, this study validated the model's stability. Integrating probability ranking and effect size estimates consistently demonstrated the following conclusions: injection interventions such as PRP, HA, and CS all exhibit common therapeutic effects in improving short-term pain and function, indicating that IAI can effectively alleviate symptoms overall. NMA and SUCRA ranking results indicate that US+PRP intervention demonstrated outstanding performance across multiple outcome measures. Its SUCRA rankings place it among the top interventions in all WOMAC dimensions: VAS (41.31%), WOMAC-T (85.86%), WOMAC-P (78.55%), WOMAC-S (93.24%), and WOMAC-F (90.9%), suggesting superior therapeutic potential. These findings align with recent positive evidence from RCTs and translational studies on PRP (37, 38). Moreover, this trend corroborates multiple direct and indirect comparisons incorporated in our study, indicating a high likelihood of US+PRP becoming a preferred strategy for early-to-mid stage knee osteoarthritis. It is crucial to emphasize that this study focuses on the key technical aspect of “US-Guided” intervention: Through real-time visual localization, therapeutic agents can be precisely delivered into the joint cavity or targeted areas such as synovial plica and infrapatellar fat pad regions. This approach minimizes inadvertent infiltration into soft tissues or vasculature, thereby enhancing injection success rates and therapeutic consistency. From a methodological framework perspective, this establishes a more reliable technical foundation for authentic efficacy comparisons across different injection protocols (39, 40).

PRP is a platelet concentrate obtained by centrifuging autologous peripheral blood (41), whose primary bioactive components include high-concentration platelets, platelet-derived growth factors, transforming growth factor-β, vascular endothelial growth factor insulin-like growth factor-1, anti-inflammatory cytokines, fibrinogen, among others (42). The multiple therapeutic advantages of PRP therapy for KOA can be attributed to its enriched bioactive network and comprehensive remodeling capability on the joint microenvironment (43). Regarding anti-inflammatory mechanisms, the interleukin-1 receptor antagonist abundantly present in PRP effectively competitively inhibits IL-1β binding to its receptor, thereby blocking NF-κB signaling pathway activation and reducing the release of pro-inflammatory factors such as TNF-α and IL-6 (44). Moreover, PRP can upregulate the expression of anti-inflammatory cytokines such as IL-10 and IL-4, promoting the transition of macrophages from the M1 to M2 phenotype, thereby improving the inflammatory microenvironment within the joint (45). Regarding cartilage repair, PRP not only stimulates chondrocyte proliferation and enhances the synthesis of extracellular matrix components including collagen II and aggrecan (46), but also activates signaling pathways such as PI3K/Akt and MAPK/Erk to augment cell survival capacity while inhibiting the activity of apoptosis-related proteins like Caspase-3. More importantly, PRP possesses the ability to regulate cartilage metabolic homeostasis by downregulating matrix metalloproteinase expression while simultaneously upregulating the production of tissue inhibitors of metalloproteinases, thereby delaying degradation of the cartilage matrix (47). Pain represents a common clinical symptom in KOA patients. PRP alleviates pain by reducing local inflammatory factors and prostaglandin levels while improving the periarticular environment of synovial nerve endings, consequently attenuating the peripheral sensitization process (48), explaining the sustained improvement in pain scores. These mechanisms collectively enable PRP not only to alleviate clinical symptoms but also potentially delay the structural progression of KOA, providing a foundation for explaining its long-term efficacy advantages (49). In summary, PRP extends beyond “anti-inflammatory and analgesic effects,” likely achieving gradual structural modifications and sustained functional benefits through multiple pathways-a finding consistent with our analytical results. Despite the growing popularity of PRP therapy for OA, its specific mechanisms of action on knee joint tissues remain unclear. A major issue with PRP is the lack of standardization (50), resulting in inconsistencies across studies. Significant variations in platelet counts among individual patients, combined with differences in preparation methods, lead to variable therapeutic outcomes of PRP therapy (51).

Additionally, both HA and CS demonstrated favorable efficacy in the NMA and SUCRA ranking. Particularly, US+HA showed statistically significant superiority over US+O3 in pain relief, with a VAS score reduction of −1.48 (−2.72, −0.24), indicating HA's substantial therapeutic effect in alleviating pain. The therapeutic mechanism of HA primarily manifests in its unique rheological properties and biological regulatory functions (52). As a key component of synovial fluid and the cartilage matrix, high molecular weight HA significantly enhances the viscoelasticity of joint fluid, restoring its lubricating and shock-absorbing functions. This reduces the friction coefficient and impact stress during joint loading, thereby mechanically alleviating pain (53, 54). HA also interacts with cell surface receptors (such as CD44, RHAMM, and TLR) to regulate intracellular signaling pathways, inhibit the production of inflammatory mediators, and stimulate endogenous HA synthesis (55, 56). Studies demonstrate that high molecular weight HA inhibits the NF-κB signaling pathway activated by Toll-like receptors, reducing the production of prostaglandin E2 and nitric oxide, thereby exerting anti-inflammatory and anti-nociceptive effects (57). Furthermore, HA possesses antioxidant properties, scavenging oxygen free radicals and protecting chondrocytes from oxidative stress-induced damage (58). Notably, high molecular weight HA demonstrates superior efficacy to low molecular weight HA in maintaining chondrocyte phenotype stability (59), particularly suitable for KOA patients with obesity or excessive mechanical loading. In recent years, the application of combined PRP and HA therapy has demonstrated synergistically enhanced therapeutic potential. PRP delivers multiple growth factors and cytokines to promote tissue repair, while HA functions as a biological scaffold that prolongs the retention of active factors and provides a conducive microenvironment (60). Collectively, these agents effectively reduce the inflammatory potential of adipocytes in the infrapatellar fat pad (IFP) synovial adipose tissue by downregulating the expression of pro-inflammatory cytokines and adipokines (61, 62). Clinical trials conducted by researchers confirm that the combined use of PRP and HA yields superior efficacy compared to monotherapy with either agent alone (63). However, this combined strategy currently faces challenges including insufficient standardization of formulations, procedural complexity, and relatively high treatment costs. Its widespread clinical application still requires further high-quality studies to provide robust evidence.

As a classic anti-inflammatory agent, CS primarily provides rapid symptomatic relief through potent anti-inflammatory effects and are mainly used for short-term symptom control in KOA management (64). When exogenous CS are injected into the joint cavity, they primarily bind to glucocorticoid receptors, modulate immune cell function, and regulate the expression levels of enzymes and pro-inflammatory cytokines, thereby inhibiting phospholipase A_2_ activity reduces the generation of inflammatory mediators such as prostaglandins and leukotrienes, and effectively alleviates synovitis by inhibiting the expression of inflammatory factors including IL-1 and TNF-α (65). Clinically, corticosteroid injections can rapidly relieve joint pain and improve joint function, demonstrating particularly significant efficacy in patients experiencing acute symptom exacerbation (66).

However, although corticosteroids provide pain relief comparable to PRP in the short term, long-term follow-up data generally indicate that their therapeutic effects cannot be sustained and may even adversely affect cartilage metabolism (67, 68). A randomized controlled trial demonstrated that knees receiving intra-articular triamcinolone injections every 3 months exhibited more significant cartilage loss compared to those receiving saline injections (27). Furthermore, long-term or repeated glucocorticoid administration suppresses chondrocyte proliferation, reduces collagen and proteoglycan synthesis, and accelerates cartilage matrix degradation, which likely contributes to its suboptimal long-term efficacy.

This NMA confirms that US-Guided IAI of PRP, HA, and CS effectively alleviate pain and improve function in early-to-mid-stage KOA (69). US-guided techniques significantly enhance injection precision and therapeutic reliability, offering an evidence base for selecting individualized treatment strategies in KOA. However, this study is not without limitations. Although we strictly adhered to inclusion criteria, methodological quality variations existed among the included RCTs, particularly as early studies commonly lacked blinding and allocation concealment designs, potentially introducing performance bias. Secondly, most studies featured limited sample sizes and short follow-up periods, constraining our assessment of the long-term efficacy of interventional approaches. Methodologically, the NMA revealed a lack of direct comparative evidence between interventions such as PRP vs. O_3_ and HA vs. CS, primarily relying on indirect estimates. Although the node-Splitting Method detected no significant inconsistency, the sparse network structure may compromise statistical power. Furthermore, the heterogeneity in clinical practice deserves attention: variations in US-guided injection accuracy, pharmaceutical preparation standards, and injection protocols may impact the generalizability of findings. Future research should address current evidence gaps by conducting more high-quality RCTs with rigorous designs and adequate sample sizes. Extending follow-up periods will systematically evaluate the long-term efficacy and safety profiles of interventional approaches, while simultaneously considering the balance between clinical outcomes and cost-effectiveness. Although these findings hold certain reference value, clinicians should comprehensively consider individual patient characteristics when making treatment decisions, and further high-quality research is needed for validation.

## Data Availability

The original contributions presented in the study are included in the article/Supplementary material, further inquiries can be directed to the corresponding author.
