# Supplementary material for: Efficacy of ultrasound-guided intra-articular injection in the treatment of knee osteoarthritis in early and middle stages: a network meta-analysis
Source: Front Med (Lausanne). 2025 Nov 6;12:1700950. doi: 10.3389/fmed.2025.1700950 (PMC12633648; doi:10.3389/fmed.2025.1700950)
Supplement: Supplementary file 1 [file Supplementary_file_1.docx]

Table.1 Basic Characteristics of Included Studies

| Incorporate literature | survey region | Number of patients （T/C） | Male/female(example) | | Average age (years) | | Intervention measure | | Injection frequency | Treatment cycle | Outcome index |
| --- | --- | --- | --- | --- | --- | --- | --- | --- | --- | --- | --- |
|  |  |  | T | C | T | C | T | C |  |  |  |
| Brian J. Cole - 2017 | America | 49/50 | 28/21 | 20/30 | 55.9±10.4 | 56.8±10.5 | US + PRP | US + HA | Once a week for three weeks | 3W | 1.2 |
| Timothy E. McAlindon - 2017 | America | 70/70 | 33/37 | 32/38 | 59.1±8.3 | 57.2±7.6 | US + CS | US + PL | Once every 12 weeks, 8 times in total. | 104W | 1 |
| Hamada S. AHMAD-2018 | Egypt | 45/44 | 14/31 | 14/30 | 56.2±6.8 | 56.8±7.4 | US + PRP | US + HA | Once every 2 weeks, 3 times in total | 4W | 1 |
| Arash Babaei-Ghazani-2018 | Iran | 31/31 | 7/24 | 3/28 | 59.65±10.249 | 56.26±7.887 | US + O₃ | US + CS | Single injection | 1W | 1 |
| Marie Laure Louis - 2017 | France | 24/24 | 14/10 | 11/13 | 53.2 ± 11.7 | 48.5 ± 11.5 | US + PRP | US + HA | Single injection | 12W | 1.2 |
| Bahram Naderi Nabi-2018 | Iran | 34/33 | 7/27 | 5/28 | 58.55±8.79 | 59.09±7.79 | US + PRP | US + CS | Once every 4 weeks, 3 times in total. | 12W | 1 |
| Poupak Rahimzadeh-2018 | Iran | 21/21 | 10/11 | 11/10 | 65.5±6.64 | 64.3±5.31 | US + PRP | US + DX | Once every 4 weeks, 2 times in total. | 24W | 2.3.4.5 |
| Yunfeng Zhang - 2018 | China | 30/30 | 14/16 | 12/18 | 65.45±5.76 | 66.23±4.87 | US + PRP | US + HA | Once a week, 4 times in total. | 4W | 1 |
| Arash Babaei - Ghazani - 2019 | Iran | 16/16 | 4/12 | 3/13 | 58.50±9.596 | 60.06±9.382 | US + CS | US + O₃ | Single injection | 13W | 1 |
| Sefa Gümrük Aslan-2024 | Türkiye | 49/47 | 8/41 | 5/42 | 62.59±9.52 | 62.51±8.59 | US + O₃ | US + CS | T: once a week, three times in total. C: single injection | T：3W C：1W | 1 |
| Murillo Dório et al. 2021 | Brazil | 21/21 | 2/19 | 2/19 | 66.1±7.5 | 62.5±8.1 | US + PRP | US + PL | Once every 2 weeks, 2 times in total. | 24W | 1.2.3.4.5 |
| Michael Baria, 2022 | America | 30/28 | 20/10 | 8/20 | 51.9±2.4 | 56.1±1.7 | US + PRP | US + AAT | Single injection | 1W | 1 |
| Michael Baria-2024 | America | 23/26 | 16/7 | 8/18 | 52.8±14.0 | 56.7±7.8 | US + PRP | US + AAT | Single injection | 52W | 1 |
| Alireza Teymouri-2025 | Iran | 25/25 | 8/17 | 7/18 | 63.24±5.71 | 62.72±5.12 | US + DX | US + PL | Single injection | 1W | 2.3.4.5 |

Table note：Outcome index:1 .VAS, 2 .WOMAC-P, 3 .WOMAC-F, 4 .WOMAC-S, 5 .WOMAC-T.

Table.2 Model Fit and Heterogeneity Evaluation of Consistency Models Across Outcomes

| Outcome index | types of models | DIC | D̄ | pD | I² (%) | PSRF（maximum） | Convergence judgment | model select |
| --- | --- | --- | --- | --- | --- | --- | --- | --- |
| VAS | Consistency+Fixed effect | 139.68 | 134.68 | 5.00 | 92 | 1.00 | Convergent | Consistency+random effect model |
|  | Consistency +Random effect | 24.07 | 12.22 | 11.85 | 10 | 1.00 | Convergent |  |
|  | Inconsistency+Fixed effect | 138.63 | 132.63 | 6.00 | 92 | 1.00 | Convergent |  |
|  | Inconsistency+Random effect | 24.06 | 12.17 | 11.89 | 10 | 1.00 | Convergent |  |
| WOMAC-P | Consistency+Fixed effect | 14.77 | 11.77 | 3.00 | 66 | 1.00 | Convergent | Consistency+random effect model |
|  | Consistency+Random effect | 10.28 | 5.50 | 4.78 | 27 | 1.00 | Convergent |  |
|  | Inconsistency+Fixed effect | 8.02 | 4.02 | 4.00 | 0.4 | 1.00 | Convergent |  |
|  | Inconsistency+Random effect | 9.00 | 4.50 | 4.50 | 11 | 1.00 | Convergent |  |
| WOMAC-F | Consistency+Fixed effect | 6.55 | 4.55 | 2.0 | 56 | 1.00 | Convergent | Consistency+random effect model |
|  | Consistency+Random effect | 5.99 | 3.25 | 2.74 | 38 | 1.00 | Convergent |  |
|  | Inconsistency+Fixed effect | 5.99 | 3.00 | 3.00 | 33 | 1.00 | Convergent |  |
|  | Inconsistency+Random effect | 5.99 | 2.99 | 2.99 | 33 | 1.00 | Convergent |  |
| WOMAC-S | Consistency+Fixed effect | 5.32 | 3.32 | 2.00 | 40 | 1.00 | Convergent | Consistency+random effect model |
|  | Consistency+Randomeffect | 5.54 | 2.94 | 2.60 | 32 | 1.00 | Convergent |  |
|  | Inconsistency+Fixed effect | 6.00 | 3.00 | 3.00 | 33 | 1.00 | Convergent |  |
|  | Inconsistency+Random effect | 5.98 | 2.99 | 2.99 | 33 | 1.00 | Convergent |  |
| WOMAC-T | Consistency+Fixedeffect | 14.69 | 12.69 | 1.99 | 84 | 1.00 | Convergent | Consistency+random effect model |
|  | Consistency+Random effect | 6.35 | 3.32 | 3.03 | 40 | 1.00 | Convergent |  |
|  | Inconsistency+Fixed effect | 6.00 | 3.00 | 3.00 | 33 | 1.00 | Convergent |  |
|  | Inconsistency+Random effect | 6.00 | 3.00 | 3.00 | 33 | 1.00 | Convergent |  |

Table note：DIC was used to comprehensively evaluate model goodness-of-fit and complexity, with lower values indicating better model performance; D represents the posterior mean of the deviance, reflecting the overall fitting error; pD denotes the effective number of parameters, serving as a measure of model complexity; I² is a heterogeneity metric, representing the proportion of observed variance attributable to non-random factors-higher I² values indicate greater variability among studies; PSRF was applied to assess the convergence of MCMC simulations, with values close to 1 indicating sufficient model convergence.

Table.3 List of Abbreviations

| Abbreviation | Full Term |
| --- | --- |
| KOA | Knee Osteoarthritis |
| TKA | Total Knee Arthroplasty |
| NSAIDs | nonsteroidal anti-inflammatory drugs |
| IAI | Intra-articular Injection |
| HA | Hyaluronic Acid |
| CS | Corticosteroids |
| PRP | Platelet-Rich Plasma |
| US | Ultrasound |
| PL | placebo |
| AAT | Autologous adipose tissue |
| O_3_ | Ozone |
| NMA | Network Meta-Analysis |
| BNMA | Bayesian Network Meta-Analysis |
| RCT | Randomized Controlled Trial |
| VAS | Visual Analogue Scale |
| WOMAC | Western Ontario and McMaster Universities Osteoarthritis Index |
| SMD | Standardized Mean Difference |
| CI | Confidence Interval |
| DIC | Deviance Information Criterion |
| PSRF | Potential Scale Reduction Factor |
| SUCRA | Surface Under the Cumulative Ranking Curve |
| IFP | infrapatellar fat pad |
